# Evaluating Biological Properties of Stingless Bee Propolis

**DOI:** 10.3390/foods12122290

**Published:** 2023-06-06

**Authors:** Jin Ru Lim, Lee Suan Chua, Dawood Ali Salim Dawood

**Affiliations:** 1 Institute of Bioproduct Development, Universiti Teknologi Malaysia, Skudai, Johor Bahru 81310, Johor, Malaysia; jinru94@gmail.com; 2 Department of Bioprocess and Polymer Engineering, School of Chemical and Energy Engineering, Faculty of Engineering, Universiti Teknologi Malaysia, Skudai, Johor Bahru 81310, Johor, Malaysia; 3 Department of Pathology and Forensic Medicine, Collage of Medicine, Wasit University, Kut 52001, Iraq; alisalim@uowasit.edu.iq

**Keywords:** chemical composition, maceration, stingless bee propolis, ultrasonic pretreatment, antioxidant, antibacterial activity, anticancer

## Abstract

The aim of the present study was to determine the content of phenolics, flavonoids and tannins, as well as the biological functions of propolis extracts from the stingless bee (*Heterotrigona itama*). The raw propolis was extracted via maceration with ultrasonic pretreatment in 100% water and 20% ethanol. The yield of ethanolic propolis extracts was about 1% higher than its aqueous counterpart. The colorimetric assays showed that the ethanolic propolis extract had about two times higher phenolics (17.043 mg GAE/g) and tannins (5.411 mg GAE/g), and four times higher flavonoids (0.83 mg QE/g). The higher phenolic content had enhanced the antiradical and antibacterial capacities of the ethanolic extract. The propolis extracts significantly exhibited higher antibacterial activity against gram-positive bacteria (*Staphylococcus aureus*) than gram-negative bacteria (*Escherichia coli* and *Pseudomonas aeruginosa*). However, aqueous extract was found to have a higher anticancer property based on the viability of lung cancer cells. No cytotoxic effect was observed on normal lung cells as the cell viability was maintained >50%, even the concentration of propolis extracts were increased up to 800 µg/mL. Different chemical compositions of propolis extract would show different bioactivities depending upon the individual applications. The high content of phenolics suggests that the propolis extract could be a natural source of bioactive ingredients for the development of innovative and functional foods.

## 1. Introduction

Meliponiculture is a stingless bee keeping activity whereby beekeepers maintain, propagate and utilize the colonies of stingless bees for profits from the harvested honey, pollen, cerumen and propolis [1,2]. It can be regarded as a potential alternative to apiculture, especially in the regions where stingless bees are endemic. Stingless bees (Hymenoptera, Apidae: Meliponini) are the largest and the most diverse corbiculate eusocial bees, which represent more than 60 genera and 600 species [3]. They are widely distributed in the warm and humid environment of tropical and subtropical forests in Malaysia. Such bees are different from the genus Apis in terms of morphology (lack of sting), nectar collection, short harvesting distance in hunting for foods and honeycomb-free colonies [4] The common stingless bee species are *Heterotrigona itama*, *Geniotrigona thoracica*, *Lepidotrigona terminata*, *Tetrigona apicalis* and *Homotrigona alicae* in Malaysia. Of these species, *Heterotrigona itama* is the commonly domesticated species in Malaysia [5].

The word “propolis”, comes from the Greek “pro” (in defense of) and polis (city/community), which means “natural product in defense of the community” [6]. Propolis, which is also known as bee glue, is a resinous natural substance from beehives. The resinous material is derived from flower buds, plant leaves and crack barks combined with wax and salivary enzymes of bees [7]. It is used to heal hive cracks and damage, as well as to protect the beehive from insect predation and microbial invasion [8]. It is sticky at and above room temperature (20 °C), but it becomes hard and brittle at lower temperature [9]. Propolis is variable in terms of physical appearance and chemical properties, depending upon botanical and geographical origins [10].

The utilization of propolis as a traditional folk medicine has been developed by various ancient populations over millennia. The historical record showed that ancient Egyptians, Greeks, Persians and Romans used propolis for the treatment of various ailments such as ulcers and internal and external wounds [3]. The therapeutic benefits of propolis have been scientifically confirmed by scientific studies. Numerous studies reported that propolis showed several biological activities including antioxidants [11], antibacterial [12], anti-hyperglycemic [13], antifungal [14] and cytotoxicity effect in some human cancer cell lines [15] in recent decades. The biological activities were contributed to by the presence of bioactive compounds in propolis extracts. According to Abdullah et al. [8], the nutritional compositions of *H. itama* propolis from Brunei was approximately 0.30% crude fiber, 45.60% total lipids, 0.43% total carbohydrates and 0.18% crude protein. Nna and co-workers [13] reported that propolis consisted of chemical compounds such as alkaloids, flavonoids, phenols, tannins, saponins, terpenoids, resins, glycosides and xanthoproteins. Many minerals such as potassium, magnesium and sodium were also detected in the propolis of stingless bee [12].

Propolis has been widely extracted using different extraction techniques which were optimized to obtain high yield and good quality of propolis extract. It was found that maceration was widely used as the conventional technique for propolis extraction [15,16,17]. The preference of this technique is mainly due to its simple and non-thermal process in order to avoid any degradation of heat-sensitive compounds. The study reported by Nna et al. [13] and Laaroussi et al. [16] showed that raw propolis macerated for 7 days could produce a higher yield of phenolic content. The modern techniques that have been used to extract propolis include ultrasound assisted extraction [18], supercritical CO_2_ extraction [19] and microwave-assisted extraction [20]. Supercritical CO_2_ is non-polar solvent, thus having a low extraction capacity for polar components such as phenolic acids and flavonoids, which are the key chemical constituents in propolis [21]. Ultrasound-assisted extraction (UAE) has the ability to overcome the disadvantages of conventional extraction for low yield and high extraction time. Oroian et al. [22] reported that 30 min was the optimum time of propolis extract using UAE, particularly to obtain higher yield of phenolic acids and flavonoids. A few studies also reported that microwave-assisted extraction of propolis showed to have lower yield of phenolic compounds than the technique of UAE for propolis [20,23]. Nevertheless, the performance of extraction is also strongly dependent on the extraction solvent. Suárez et al. [24] reported that ethanolic propolis extraction could produce the highest extraction yield compared to other organic solvents such as methanol, chloroform, hexane and ethyl acetate. Before the work of Suarez et al. [24], there were also studies that revealed that aqueous ethanolic solution was the most commonly used solvent for the extraction of bioactive compounds from propolis [11,20]. In particular, the bioactive compounds were mostly from the class of phenolics and flavonoids [22,25].

Although many studies on propolis are available, such investigation for stingless bee propolis in Malaysia is relatively limited in literature. Therefore, the aim of this study was to compare the quality of propolis extracts from *H. itama* using maceration with ultrasonic pretreatment in both 100% water and 20% ethanol as the solvent systems. The comparison was focused on the total phenolic, flavonoids and tannins, as well as the antioxidant, antibacterial and anticancer properties of propolis extracts. A good quality of propolis extract with known biological properties is likely to be a potential bioactive ingredient for the development of nutraceutical and functional foods. Furthermore, valorization of meliponiculture waste into valuable ingredient is important for the sustainability of economic growth.

## 2. Materials and Methods

### 2.1. Sample Collection

The raw propolis of *Heterotrigona itama* was harvested in the month of October 2022 from a stingless bee farm in Senai, Johor. The raw propolis was stored in a refrigerator overnight at 4 °C prior to cutting and extraction. The raw propolis was manually cut into small pieces of about 0.5 to 1.0 cm.

### 2.2. Reagents and Chemicals

Methanol, sodium carbonate and Folin–Ciocalteu phenol reagent were purchased from Merck (Gernsheim, Darmstadt, Germany). Ethanol was purchased from Fisher Scientific (Pittsburg, CA, USA). Casein (from bovine milk), 2,2-diphenyl-1-picrylhyfrazyl (DPPH), fluorescein (free acid, dye content 95%), Muller Hinton Broth (powder form), Tryptic Soy Agar and thiazolyl blue tetrazolium bromide (98%) were obtained from Sigma-Aldrich (St. Louis, MO, USA). Aluminum chloride, potassium dihydrogen phosphate and potassium hydrogen phosphate were sourced from Qrec (New Zealand). 2,2′-azobis(2-methylpropionamidine) dihydrochloride (AAPH, 98%) was purchased from Acros Organics (Geel, Belgium). All chemicals were of analytical grade unless otherwise stated.

The standard chemicals such as gallic acid (97.5%), quercetin (95%), 6-hydroxy-2,5,7,8-tetramethylchroman-2-carboxylic acid (Trolox, 97%), polymyxin B (20 mg/mL in water) and vancomycin (≥900 μg/mg) were purchased from Sigma-Aldrich (St. Louis, MO, USA). Ascorbic acid (97%) was obtained from Merck (Gernsheim, Darmstadt, Germany).

Human adenocarcinoma cell line (A549; CCL-185™) and human lung fibroblasts cell line (MRC-5; CCL-171™) were purchased from American Type Culture Collection (ATCC, Manassas, VA, USA). The standard bacterial strain, *Escherichia coli* (ATCC 25922), *Pseudomonas aeruginosa* (ATCC 9027) and *Staphylococcus aureus* (ATCC 25923) were purchased from American Type Culture Collection (Manassas, VA, USA).

Roswell Park Memorial Institute 1640 (RPMI 1640), Dulbecco’s Modified Eagle Medium (DMEM), fetal bovine serum (FBS) and penicillin (10,000 U/mL)-streptomycin (10,000 μg/mL) were sourced from Thermo Fisher Scientific (Waltham, MA, USA).

### 2.3. Maceration of Propolis with Ultrasonic Pretreatment

Two sets of cut raw propolis samples (100 g) were prepared and topped with 100% distilled water (1 L) and 20% ethanol (1 L) in two different Schott bottles, separately. The mixtures were subjected to 30 min of ultrasonic pretreatment at 25 °C using an ultrasonicator (WUC-D, Daihan Scientific, Wonju, Republic of Korea). The samples were left for maceration for 7 days at 25 °C after the treatment. Similarly, the mixture was filtered, and the supernatant was concentrated by a rotary evaporator (4001, Heidolph, Schwabach, Germany) at 40 °C. The concentrated propolis extracts were then further dried in an oven at 40 °C, and the dried propolis extracts were kept at 4 °C until further analyses.

### 2.4. Total Phenolic Content

The total phenolic content (TPC) of propolis extracts was determined according to the procedure explained by Ahn and co-workers [26] with some modification. Folin–Ciocalteu reagent was used to measure the color change upon the reaction with phenolic compounds in samples. A 0.5 mL sample (1–3 mg/mL) was mixed with 0.5 mL Folin–Ciocalteu reagent and 0.5 mL sodium carbonate (10% *w/v*). Distilled water was then added to bring the total volume of the mixture to 5.0 mL. An UV–VIS spectrophotometer (UV-1800, Shimadzu, Kyoto, Japan) was used to measure the absorbance of the mixture at 760 nm after the mixture was incubated for 1 h at 25 °C. Gallic acid (1 to 5 mg/L) was prepared and used as the standard chemical for the construction of calibration curve. The results are expressed in milligram gallic acid equivalent per gram of propolis extract (mg GAE/g).

### 2.5. Total Flavonoid Content

The total flavonoid content (TFC) was determined using the colorimetric aluminum chloride method in accordance with the procedure outlined by Abduh and co-authors [27]. A 2.0 mL sample (1–3 mg/mL) was mixed with 3 mL methanolic aluminum chloride (5% *w/v*) and incubated at 25 °C for 30 min. The absorbance was measured at 415 nm using the UV–VIS spectrophotometer. In this experiment, quercetin (1 to 5 mg/L) served as the standard chemical. The results are expressed in milligram quercetin equivalent per gram of propolis extract (mg QE/g).

### 2.6. Total Tannin Content

The method of casein precipitation, which was published by Monteiro et al. [28], was slightly modified to determine the total tannin content (TTC) of propolis extracts. Casein was used to precipitate tannin phenolics and left non-tannin phenolics in the solution. In this assay, a 6.0 mL sample (1–3 mg/mL) was mixed with 1 g of casein and 12 mL of distilled water. The mixture was agitated for 3 h at 25 °C, and the supernatant was then collected by filtration. The Folin–Ciocalteu reagent was then used to determine the TPC in the filtrate (liquid phase) based on the above-mentioned TPC assay. TTC is equal to the difference of TPC in propolis extract and TPC in the filtrate (non-tannin phenolics) as presented in Equation (1).

Total tannin content (mg/g) = Total phenolic content (sample) − Total phenolic content (filtrate)(1)

### 2.7. DPPH Radical Scavenging Assay

The free radical scavenging capacity of propolis extracts was determined using a DPPH (2,2-diphenyl-1-picrylhydrazyl) assay according to the method described by Saidan et al. [29]. A 1.0 mL sample solution (20–1000 mg/L) was mixed with 1.0 mL of methanolic DPPH solution (0.1 mM). The absorbance of the mixture was measured at 517 nm using an UV–VIS spectrophotometer (UV-1800, Shimadzu, Japan) after 30 min of incubation in the dark place. Ascorbic acid solution (1–5 mg/L) was prepared and used as the standard chemical to construct a calibration curve. The inhibition of propolis samples can be calculated using Equation (2).

(2) $$\mathrm{Inhibition}\;(\%)=\frac{Ab-Aa}{Ab}\times 100$$

where *Ab* is the absorption of blank, while *Aa* is the absorption of sample. The results are expressed in IC_50_, which is the required concentration of sample to reduce 50% of the inhibitory percentage.

### 2.8. Oxygen Radical Absorption Capacity

The absorption capacity of oxygen radical (ORAC) was determined according to the procedures described by Andrade et al. [30] with modification. A 1.50 mL fluorescein working solution (6.30 mmol/L) and 0.75 mL samples (1.0–3.0 mg/mL) or standard Trolox (2.0–10.0 mg/L) were mixed and incubated at 37 °C for 15 min. Then, 0.75 mL of AAPH working solution (153 mmol/L) was added into the mixture. The absorbance was monitored at 90 s interval for 1 h using the spectrophotometer (UV-1800, Shimadzu, Japan) at 520 nm. The ORAC value can be calculated by subtracting the reaction curve area of the blank with the reaction curve area of the sample. The results are expressed in milligram Trolox equivalent per g propolis (mg TE/g).

### 2.9. Antibacterial Assay

The water and ethanolic propolis extracts from ultrasonic pretreated maceration were further studied for their antibacterial performance. The in vitro antibacterial property of propolis extracts was tested against three pathogenic microorganisms such as *E. coli*, *P. aeruginosa* and *S. aureus*. The bacterial strains were cultured on tryptone soya agar for one day before use. The bacterial suspensions were adjusted to McFarland 0.5 with approximately 1.50 × 10^8^ cfu and then further diluted to approximately 5.0 × 10^5^ cfu/mL for inoculation.

The technique of microdilution was used to determine the minimum inhibitory concentration (MIC) and minimum bactericidal concentration (MBC). The assay was performed following the method described in M07 [31]. The concentration of water extracts was prepared in the range of 0.13 to 267.75 mg/g and the ethanolic extracts were ranged from 0.16 to 320.75 mg/g by serial dilution. The concentration of antibiotic ranged from 0.0122 to 25 µg/g.

Muller Hinton Broth (100 µL) was added into 96-well microplates and then 100 µL of sample or antibiotic was added in triplicate. A two-fold dilution was carried out. Bacterial strain (100 µL) was added into each well and the plate was incubated for 24 h at 35 °C. Each bacterial strain treated with different concentrations of samples was streaked onto tryptic soy agar plates and further incubated for 24 h to determine the minimum bactericidal concentration (MBC). A 50 µL MTT reagent was added into wells and re-incubated for 2 h to observe the cell viability based on the color change from yellow colored tetrazolium salt to insoluble purple colored formazan. The minimum inhibitory concentration (MIC) was determined from the color change.

### 2.10. Cultivation of Cancer Cell Lines

Human adenocarcinoma cell line (A549; CCL-185™) and human lung fibroblasts cell line (MRC-5; CCL-171™) were purchased from American Type Culture Collection (ATCC, Rockville, MD, USA). The A549 cell line was maintained in Roswell Park Memorial Institute-1640 (RPMI-1640) medium, while the MRC-5 cell line was cultured in Modified Eagle Medium (MEM). Both mediums were supplemented with 10% fetal bovine serum (FBS), 100 U/mL penicillin and 100 μg/mL streptomycin at 37 °C with 5% CO_2_.

### 2.11. Cell Viability

The viability of treated cells was determined using a 3-[4,5-dimethylthiazol-2-yl]-2,5 diphenyltetrazolium bromide assay (MTT). Briefly, (1 × 10^4^ cells/well) of A549 and MRC-5 were seeded in 96-well plates and treated with various concentrations (50 to 800 µg/mL) of samples and cisplatin (positive control) for 24 h, while DMSO was used as a negative control in this assay. A 20 μL of MTT solution was added to each well and incubated in a humidified 5% CO_2_ incubator at 37 °C for 4 h. Then, 100 μL of (DMSO) was added to solubilize the formazan crystals. The absorbance was measured at 570 nm using an ELISA microplate reader (Bio-Rad, Hercules, CA, USA).

### 2.12. Statistical Analysis

One-way ANOVA followed by *t*-test was used to analyze the experimental data to determine whether the mean values of the results were significantly different (*p* < 0.05) at 95% confidence level. The software used for the statistical analysis was Excel^®^ 2016 (Microsoft Corporation, Redmond, WA, USA).

## 3. Results and Discussion

### 3.1. Extraction of Propolis

Extraction is the main process for the isolation and recovery of compounds from natural product samples. The variation of sample size, pH, stirring speed, temperature, solvent and sample to solvent ratio play an important role in affecting the yield of extraction. One of the most influential extraction variables was the solvent composition [32,33]. The solvent system of 100% water and 20% ethanol was used to extract propolis from the raw material using maceration with ultrasonic pretreatment in this study. Ultrasound is a sound wave which can produce cavitation bubbles within liquid. The generated bubbles would increase in size over the period of extraction time and then collapse to generate energy, which would consequently increase the pressure in the extraction medium. The sample structure would be destroyed under the increased pressure environment. This phenomenon would promote the penetration of the solvent into the sample to release compounds into the solvent [34]. A short period of ultrasonic pretreatment (30 min) most probably assisted the diffusion of compounds from resinous raw propolis resulted from the breakage of its polymeric structure.

**Figure 1:**
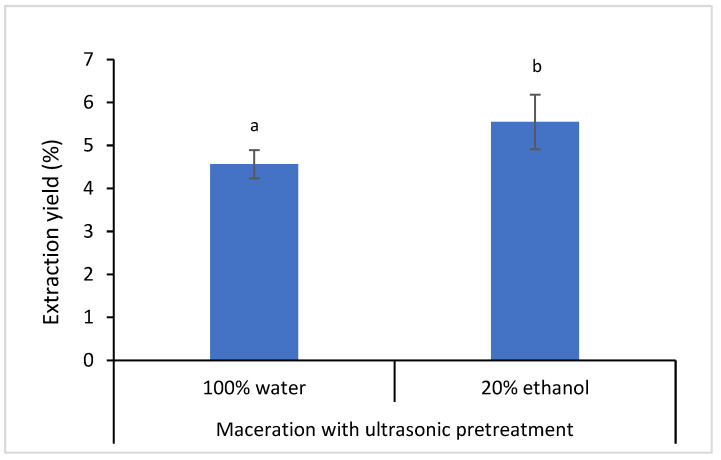
The extraction yield of propolis extracts obtained from different solvents. Different letters indicate that the mean of data is significantly different at *p* < 0.05.

Figure 1 shows the results of extraction yield, which is about 1% difference between the aqueous and ethanolic propolis extracts. The solvent system which consisted of water (80%) and ethanol (20%) could produce a higher yield of propolis extract, mainly both water soluble and ethanol soluble compounds could diffuse into the solvent medium. The solvent system with the higher composition of water was chosen since propolis mostly contains phenolic acids and hydrolyzable tannins which are water soluble [35]. A high percentage of ethanol (60–80%) was usually used as the solvent system to extract polyphenol-rich leaves from plant samples [36,37]. Water is used to soften the structure of raw propolis, whereas ethanol is a favorable solvent for the extraction of phenolic compounds. Previous studies reported that ethanol was likely an effective solvent for polyphenol extraction [32].

### 3.2. Total Phenolic, Flavonoid and Tannin Content

**Figure 2:**
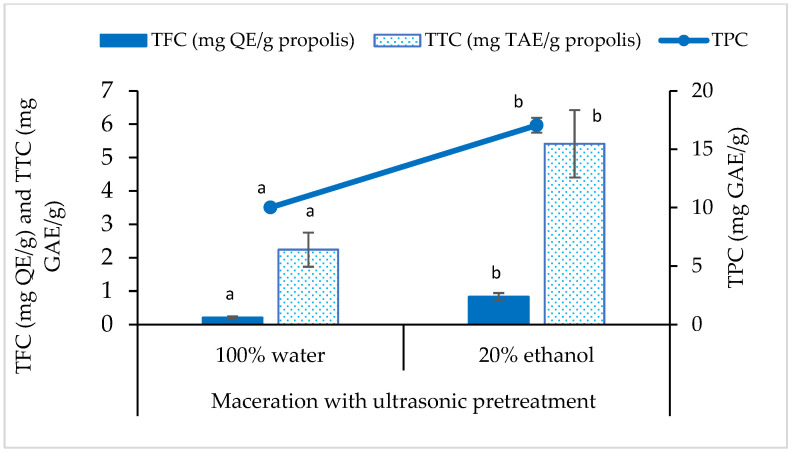
The total phenolic content (TPC, solid line), total flavonoid content (TFC, solid bar) and total tannin content (TTC, dot bar) of propolis extracts using different solvents. Different letters indicate that the values are significantly different in the same test (*p* < 0.05).

Phenolics consist of a broad range of compounds, mostly tannins, flavonoids and phenolic acids. As shown in Figure 2, the propolis extracts showed to have the lowest flavonoid content, followed by the content of tannins and phenolics. It was about less than 0.1% flavonoids, 0.2–0.5% tannins and 1.0–1.7% phenolics in the propolis extracts. A slight increment was observed when 20% ethanol was added into the solvent system for extraction. Based on the total content, flavonoids and tannins were approximately covered for 3.1% to 11.3% and 22.4% to 31.7% of TPC, respectively.

The results of TPC (10.017 to 17.043 mgGAE/g) and TFC (0.204 to 0.830 mgQE/g) in the present study were higher than the data reported by other researchers from Malaysia (TPC = ~0.697 µgGAE/g and TFC = ~7.437 µgQE/g) [38]. However, the results were lower than the data reported by researchers from Brunei (TPC = 2192.7 to 2391.0 mgGAE/g and TFC = 275.2 to 299.4 mgQE/g) [12] and Indonesia (TPC = 98.082 mgGAE/g and TFC = 15.890 mgQE/g) [39]. They also sourced their propolis samples from stingless bees, but they performed different extraction methods using a higher concentration of ethanol (80–96%). Previous findings showed that the variation of phenolic and flavonoid content could possibly be contributed by multiple factors such as the difference of extraction techniques and solvent system, in addition to plant preference and vegetation by bees [40].

The total content of tannins in propolis is seldom discussed in literature. Mayworm et al. [41] reported that the detection of tannins ranged from 0.6 to 4.1% in propolis samples collected from different regions of Brazil. The value range was higher than the data reported in the present study (0.2 to 0.5%). Tannins are water soluble polyphenols, and they tend to precipitate with protein (casein). Hence, its content can be estimated from the method of protein precipitation with casein. The result also recorded that TTC was positively correlated with TPC, since TTC is part of the TPC. According to Kiziltas and Erkan [42], the dark color of propolis extract was most probably attributed by tannins. Hence, tannins found in propolis extracts might be mostly contributed by woody plant of jaboticaba (Myrtaceae) plantation where the raw material of propolis was harvested.

### 3.3. Antioxidant Capacity of Propolis Extracts

Free radicals can be deleterious to cells and lead to various chronic diseases such as diabetes, arthritis and asthma [18]. The radical scavenging capacity of stingless bee propolis extracts was determined using the colorimetric assays of DPPH and ORAC. Table 1 presents the results of DPPH and ORAC of propolis extracts. In the DPPH assay, the effective concentration of propolis extracts to achieve 50% inhibition (IC_50_) were 96.28 mg/g and 30.77 mg/g for 100% water and 20% ethanol propolis extracts, respectively. The lower IC_50_ indicated higher antiradical capacity of propolis extracted from 20% ethanol. Similarly, the performance of ethanolic extract was better to scavenge peroxyl radicals as observed in ORAC assay. Ethanolic propolis extract could achieve higher Trolox equivalent. In line with the results of proximate chemical composition, the antioxidant capacity of propolis extract was mainly attributed to the presence of flavonoids, tannins and phenolics.

The antioxidant capacity of stingless bees propolis extracts measured by the DPPH method in the present study (0.323 to 0.664 mg/mL) was comparable with stingless bee propolis extracts from Thai (0.122 to 1.228 mg/mL) [18] and Morocco (0.008 to 1.813 mg/mL) [43]. On the other hand, the antioxidant capacity of stingless bee (*H. itama*) propolis from Brunei (0.076 mg/mL) [12] and Algeria (0.022 to 0.042 mg/mL) [44] were higher with lower IC_50_ values in the DPPH assay. The variance was probably due to the differences of bee species and geographical origin. The ORAC values reported in the present study (0.249 to 15.266 µmol TE/g) were lower than the results of the ORAC reported by Miguel et al. (1106.423 to 2012.152 µmol TE/g) [43], Andrade et al. (6665 to 6734 µmol TE/g) [30] and Cavalaro et al. (21.3 to 13224 µmol TE/g) [11].

### 3.4. Antibacterial Activity

Bacterial antibiotic resistance has been accelerated over time due to the misuse of antibiotics. The utilization of new, natural and multitargeting antibacterial products may help to control the antibiotic resistance [17]. Therefore, the antibacterial potential of propolis extracts was assessed in this study.

Based on the results of chemical composition and antioxidant capacity, the ultrasonic treated propolis extracts were further examined for their antibacterial properties. Table 2 shows the MIC and MBC of the samples compared to antibiotics. The table shows gram-positive bacterium (*S. aureus)* was more susceptible than gram-negative bacteria (*E. coli* and *P. aeruginosa*) to propolis extracts. This was in agreement with previous studies which showed that propolis had higher antimicrobial activity against gram-positive bacteria than gram-negative bacteria [17,44]. The MIC of *S. aureus* was 8.36 mg/g and 5.01 mg/g using water and 20% ethanol propolis extracts, respectively. The gram-negative bacteria have an outer membrane composed of lipopolysaccharides and proteins as a protective layer against the inhibitory action of propolis extracts. Regardless the solvent system, the propolis extracts exhibited the MIC at 267.75 mg/g and 320.75 mg/g against the growth of *E. coli* and *P. aeruginosa,* respectively. Both extracts showed no MBC against all tested bacteria, even though the concentration of propolis extracts was increased up to 320.75 mg/g.

The propolis extracts showed bacteriostatic effect, but no bactericidal activity against the three tested bacteria. The bacteriostatic and bactericidal effects of propolis could be related to their ability to inhibit protein synthesis and prevent cell division [44]. Additionally, the antimicrobial potential of propolis could be attributed to the synergistic effects of phenolic compounds, which could act on the internal bacterial membrane, altering the potential and, therefore, its permeability [17]. Gram-negative bacteria are more resistant, which might be due to the more complex cell wall with an external cell membrane [17]. However, the extent of MIC is strongly influenced by the chemical composition of propolis, which is also subject to the difference of geographical and vegetation origins, as well as climate factor [45]. This was proven from different values of MIC reported for Brazil propolis (1.56 mg/g) [46] and Indonesian propolis (0.256 mg/g) [47] against the growth of *S. aureus.*

### 3.5. Anticancer Property of Propolis Extracts

**Figure 3:**
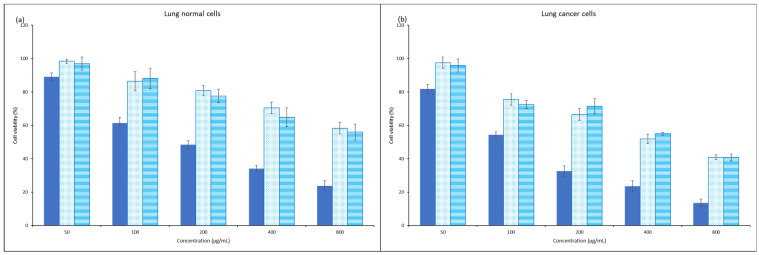
Viability of normal and cancer cells treated with different concentrations of cisplatin (solid bar) and propolis extracts (dot bar, 100% water and line bar, 20% ethanol).

The anticancer property of propolis extracts was also examined using lung cancer cells. The cell viability is plotted in a concentration dependent manner as illustrated in Figure 3. The results, which are expressed in IC_50_, are presented in Table 3. The table clearly shows no cytotoxic effect of propolis extracts on the viability of normal lung cells. The large difference of IC_50_ values between normal and cancer cells explained that propolis extract was selective enough to inhibit the growth of cancer cells, without significant effect on the growth of normal cells which could maintain more than 70% viability at the concentration up to 400 µg/mL. The selectivity of propolis was also highlighted by Guzelmeric and his co-workers [48]. The effectiveness of propolis against lung cancer cells had been proven by Algerian researchers who also reported the dual effect of propolis as antitumor and chemopreventive agents in relation with antioxidant capacity [49]. Researchers found that propolis induced apoptosis of lung cancer cells via mitochondrial-mediated pathway [50], reduction of cell adhesion by altering fibrinogen [51].

**Figure 4:**
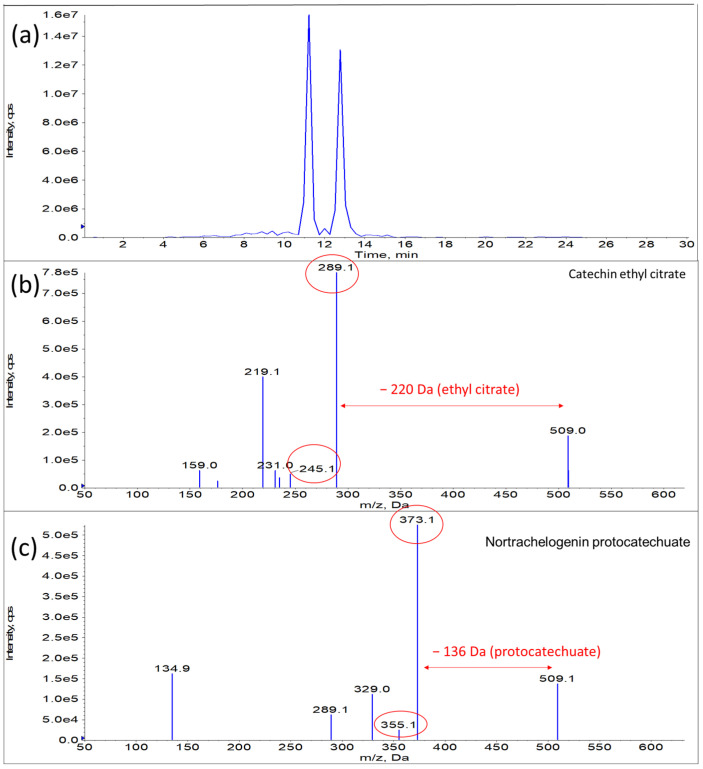
(**a**) Total ion chromatogram of compounds at m/z 509 and its mass fragments for (**b**) catechin ethyl citrate and (**c**) nortrachelogenin protocatechuate identified by mass spectrometry.

In the present study, aqueous extract could perform better to inhibit the growth of cancer cells with lower IC_50_ value. Further investigation showed that the observation could most probably be explained by the presence of metabolites such as catechin ethyl citrate (m/z 509) and nortrachelogenin protocatechuate (m/z 509) [52], which were only detected in aqueous extract, in addition to the previously reported compounds [53]. The metabolites, catechin ethyl citrate and nortrachelogenin protocatechuate had intense peaks and the similar molecular weight. The mass fragments of the compounds are illustrated in Figure 4. The compounds had been reported to have remarkable anticancer properties in previous studies [54,55]. Therefore, they could be the compounds attributed to the better inhibitory action against the growth of lung cancer cells.

## 4. Conclusions

In conclusion, a short period of ultrasonic pretreatment prior to maceration sufficiently produced propolis extracts that were rich in phenolics based on the TPC, TFC and TTC assays, especially from ethanolic propolis extract. The high content of phenolics, flavonoids and tannins were found to increase the antioxidant capacity of samples. The growth of *S. aureus* was also significantly inhibited by propolis extracts prepared by the solvent system of 20% ethanol. However, aqueous extract was found to be better to inhibit lung cancer cells. Further work is required in order to confirm chemical constituents with the antioxidative potential, which is usually a fundamental requirement for antimicrobial and anticancer properties. Bioactive propolis extract could be beneficially used as an active ingredient for innovative food and beverage products in near future.

## Figures and Tables

**Table 1:** The antioxidant activity of propolis crude extracts.

Propolis Extract	DPPH (IC_50_, mg/g)	ORAC (mg Trolox Equivalent/g Propolis)
100% water	96.28 ± 15.13	0.623 ± 0.284
20% ethanol	30.77 ± 3.17	3.821 ± 3.444

**Table 2:** Minimum inhibitory concentration of propolis extracts and antibiotics.

Samples	Minimum Inhibitory Concentration (MIC, mg/g)		
	*Escherichia coli*	*Pseudomonas aeruginosa*	*Staphylococcus aureus*
Polymyxin B (µg/g)	25	25	NA
Vancomycin (µg/g)	NA	NA	12.5
100% water	267.75	267.75	8.36
20% ethanol	320.75	320.75	5.01

NA: not applicable.

**Table 3:** Effective concentration at 50% inhibition (IC_50_) for normal and cancer cells of lung after treated with propolis extracts.

Propolis Extract	Normal Cells (µg/mL)	Cancer Cells (µg/mL)
Cisplatin	226.452 ± 7.610	153.705 ± 6.101
100% water	862.500 ± 18.128	334.516 ± 15.932
20% ethanol	669.501 ± 15.508	434.600 ± 9.281

## Data Availability

Data is contained within the article.

## References

[1] Paris E.H., Lope C.P., Masson M.A., Kú P.C.D., Ojeda B.C.E. (2018). The organization of stingless beekeeping (Meliponiculture) at Mayapán, Yucatan, Mexico. J. Anthr. Archaeol..

[2] Syafrizal, Ramadhan R., Kusuma I.W., Egra S., Shimizu K., Kanzaki M., Tangkearung E. (2020). Diversity and honey properties of stingless bees from meliponiculture in East and North Kalimantan, Indonesia. Biodiversitas J. Biol. Divers..

[3] Zulhendri F., Perera C.O., Chandrasekaran K., Ghosh A., Tandean S., Abdulah R., Herman H., Lesmana R. (2021). Propolis of stingless bees for the development of novel functional food and nutraceutical ingredients: A systematic scoping review of the experimental evidence. J. Funct. Foods.

[4] Se K.W., Ghoshal S.K., Wahab R.A., Ibrahim R.K.R., Lani M.N. (2018). A simple approach for rapid detection and quantification of adulterants in stingless bees (*Heterotrigona itama*) honey. Food Res. Int..

[5] Ismail N.F., Maulidiani M., Omar S., Zulkifli M.F., Radzi M.N.F.M., Ismail N., Jusoh A.Z., Roowi S., Yew W.M., Rudiyanto R. (2021). Classification of stingless bee honey based on species, dehumidification process and geographical origins using physicochemical and ATR-FTIR chemometric approach. J. Food Compos. Anal..

[6] Silva-Beltrán N.P., Umsza-Guez M.A., Rodrigues D.M.R., Gálvez-Ruiz J.C., Castro T.L.d.P., Balderrama-Carmona A.P. (2021). Comparison of the biological potential and chemical composition of Brazilian and Mexican propolis. Appl. Sci..

[7] Salleh S.N.A.S., Hanapiah N.A.M., Johari W.L.W., Ahmad H., Osman N.H. (2021). Analysis of bioactive compounds and chemical composition of Malaysian stingless bee propolis water extracts. Saudi J. Biol. Sci..

[8] Abdullah N.A., Ja’Afar F., Yasin H.M., Taha H., Petalcorin M.I., Mamit M.H., Kusrini E., Usman A. (2019). Physicochemical analyses, antioxidant, antibacterial, and toxicity of propolis particles produced by stingless bee *Heterotrigona itama* found in Brunei Darussalam. Heliyon.

[9] Ahmed R., Tanvir E.M., Hossen S., Afroz R., Ahmmed I., Rumpa N.-E., Paul S., Gan S.H., Sulaiman S.A., Khalil I. (2017). Antioxidant properties and cardioprotective mechanism of Malaysian propolis in rats. Evid.-Based Complement. Altern. Med..

[10] Ramli N., Ali N., Hamzah S., Yatim N. (2021). Physicochemical characteristics of liposome encapsulation of stingless bees’ propolis. Heliyon.

[11] Cavalaro R.I., da Cruz R.G., Dupont S., de Moura Bell J., Vieira T. (2019). *In vitro* and *in vivo* antioxidant properties of bioactive compounds from green propolis obtained by ultrasound-assisted extraction. Food Chem. X.

[12] Abdullah N.A., Zullkiflee N., Zaini S.N.Z., Taha H., Hashim F., Usman A. (2020). Phytochemicals, mineral contents, antioxidants, and antimicrobial activities of propolis produced by Brunei stingless bees *Geniotrigona thoracica*, *Heterotrigona itama*, and *Tetrigona binghami*. Saudi J. Biol. Sci..

[13] Nna V.U., Abu Bakar A.B., Lazin R.M.L.M., Mohamed M. (2018). Antioxidant, anti-inflammatory and synergistic anti-hyperglycemic effects of Malaysian propolis and metformin in streptozotocin–induced diabetic rats. Food Chem. Toxicol..

[14] Ibrahim M.E.E.-D., Alqurashi R.M. (2021). Anti-fungal and antioxidant properties of propolis (bee glue) extracts. Int. J. Food Microbiol..

[15] Arung E.T., Ramadhan R., Khairunnisa B., Amen Y., Matsumoto M., Nagata M., Kusuma I.W., Paramita S., Tandirogang N., Takemoto N. (2021). Cytotoxicity effect of honey, bee pollen, and propolis from seven stingless bees in some cancer cell lines. Saudi J. Biol. Sci..

[16] Laaroussi H., Ferreira-Santos P., Genisheva Z., Bakour M., Ousaaid D., Teixeira J.A., Lyoussi B. (2021). Unraveling the chemical composition, antioxidant, α-amylase and α-glucosidase inhibition of Moroccan propolis. Food Biosci..

[17] Peixoto M., Freitas A.S., Cunha A., Oliveira R., Almeida-Aguiar C. (2021). Antioxidant and antimicrobial activity of blends of propolis samples collected in different years. LWT.

[18] Vongsak B., Kongkiatpaiboon S., Jaisamut S., Machana S., Pattarapanich C. (2015). *In vitro* alpha glucosidase inhibition and free-radical scavenging activity of propolis from Thai stingless bees in mangosteen orchard. Rev. Bras. Farm..

[19] Biscaia D., Ferreira S.R. (2009). Propolis extracts obtained by low pressure methods and supercritical fluid extraction. J. Supercrit. Fluids.

[20] Pellati F., Prencipe F.P., Bertelli D., Benvenuti S. (2013). An efficient chemical analysis of phenolic acids and flavonoids in raw propolis by microwave-assisted extraction combined with high-performance liquid chromatography using the fused-core technology. J. Pharm. Biomed. Anal..

[21] Song M., Wang K., Lu H., Yan S., Wu L., Xue X. (2021). Composition and distribution of α-dicarbonyl compounds in propolis from different plant origins and extraction processing. J. Food Compos. Anal..

[22] Oroian M., Ursachi F., Dranca F. (2020). Influence of ultrasonic amplitude, temperature, time and solvent concentration on bioactive compounds extraction from propolis. Ultrason. Sonochemistry.

[23] Oroian M., Dranca F., Ursachi F. (2019). Comparative evaluation of maceration, microwave and ultrasonic-assisted extraction of phenolic compounds from propolis. J. Food Sci. Technol..

[24] Suárez G.A.P., Galindo N.J.P., Cuervo O.H.P. (2022). Obtaining Colombian propolis extracts using modern methods: A determination of its antioxidant capacity and the identification of its bioactive compounds. J. Supercrit. Fluids.

[25] Kara Y., Can Z., Kolaylı S. (2022). Applicability of phenolic profile analysis method developed with RP-HPLC-PDA to some bee product. Braz. Arch. Biol. Technol..

[26] Ahn M.-R., Kumazawa S., Usui Y., Nakamura J., Matsuka M., Zhu F., Nakayama T. (2007). Antioxidant activity and constituents of propolis collected in various areas of China. Food Chem..

[27] Abduh M.Y., Adam A., Fadhlullah M., Putra R.E., Manurung R., Abduh M.Y., Adam A., Fadhlullah M., Putra R.E., Manurung R. (2020). Production of propolis and honey from *Tetragonula laeviceps* cultivated in Modular Tetragonula Hives. Heliyon.

[28] Monteiro J.M., de Souza J.S., Neto E.M.L., Scopel K., Trindade E.F. (2014). Does total tannin content explain the use value of spontaneous medicinal plants from the Brazilian semi-arid region?. Rev. Bras. Farm..

[29] Saidan N.H., Hamil M.S.R., Memon A.H., Abdelbari M.M., Hamdan M.R., Mohd K.S., Majid A.M.S.A., Ismail Z. (2015). Selected metabolites profiling of *Orthosiphon stamineus* Benth leaves extracts combined with chemometrics analysis and correlation with biological activities. BMC Complement. Altern. Med..

[30] Andrade J.K.S., Denadai M., de Oliveira C.S., Nunes M.L., Narain N. (2017). Evaluation of bioactive compounds potential and antioxidant activity of brown, green and red propolis from Brazilian northeast region. Food Res. Int..

[31] (2018). Methods for Dilution Antimicrobial Susceptibility Tests for Bacteria that Grow Aerobically.

[32] Do Q.D., Angkawijaya A.E., Tran-Nguyen P.L., Huynh L.H., Soetaredjo F.E., Ismadji S., Ju Y.-H. (2014). Effect of extraction solvent on total phenol content, total flavonoid content, and antioxidant activity of *Limnophila aromatica*. J. Food Drug Anal..

[33] Yang L., Jiang J.-G., Li W.-F., Chen J., Wang D.-Y., Zhu L. (2009). Optimum extraction process of polyphenols from the bark of *Phyllanthus emblica* L. based on the response surface methodology. J. Sep. Sci..

[34] Xu D.-P., Li Y., Meng X., Zhou T., Zhou Y., Zheng J., Zhang J.-J., Li H.-B. (2017). Natural antioxidants in foods and medicinal plants: Extraction, assessment and resources. Int. J. Mol. Sci..

[35] Dutra R.P., de Barros Abreu B.V.d.B., Cunha M.S., Batista M.C.A., Torres L.M.B., Nascimento F.R.F., Ribeiro M.N.S., Guerra R.N.M. (2014). Phenolic acids, hydrolyzable tannins, and antioxidant activity of geopropolis from the stingless bee *Melipona fasciculata* Smith. J. Agric. Food Chem..

[36] Lau C.H., Chua L.S., Lee C.T., Aziz R. (2015). Optimization and kinetic modeling of rosmarinic acid extraction from *Orthosiphon stamineus*. Curr. Bioact. Compd..

[37] Chua L.S., Abdullah F.I., Azlah M.A.F. (2019). Phytochemical profile of *Andrographis paniculata* extract from solvent partition and precipitation. J. Biol. Act. Prod. Nat..

[38] Asem N., Gapar N.A.A., Hapit N.H.A., Omar E.A. (2019). Correlation between total phenolic and flavonoid contents with antioxidant activity of Malaysian stingless bee propolis extract. J. Apic. Res..

[39] Farida S., Pratami D.K., Sahlan M., Laksmitawati D.R., Rohmatin E., Situmorang H. (2021). In-Vitro antioxidant, in-vivo anti-inflammatory, and acute toxicity study of Indonesian propolis capsule from *Tetragonula sapiens*. Saudi J. Biol. Sci..

[40] Awang N., Ali N., Abd Majid F.A., Hamzah S., Abd Razak S.B. (2018). Total flavonoids and phenolic contents of sticky and hard propolis from 10 species of indo-malayan stingless bees. Malays. J. Anal. Sci..

[41] Mayworm M.A.S., Lima C.A., Tomba A.C.B., Fernandes-Silva C.C., Salatino M.L.F., Salatino A. (2014). Does propolis contain tannins?. Evid. Based Complement. Altern. Med..

[42] Kiziltas H., Erkan C. (2021). The effects of different beehives on propolis production and quality. Food Sci. Technol..

[43] Miguel M.D.G., Doughmi O., Aazza S., Antunes D., Lyoussi B. (2013). Antioxidant, anti-inflammatory and acetylcholinesterase inhibitory activities of propolis from different regions of Morocco. Food Sci. Biotechnol..

[44] Boulechfar S., Zellagui A., Bensouici C., Asan-Ozusaglam M., Tacer S., Hanene D. (2021). Anticholinesterase, anti-α-glucosidase, antioxidant and antimicrobial effects of four Algerian propolis. J. Food Meas. Charact..

[45] Selvaraju G.D., Umapathy V.R., SumathiJones C., Cheema M.S., Jayamani D.R., Dharani R., Sneha S., Yamuna M., Gayathiri E., Yadav S. (2022). Fabrication and characterization of surgical sutures with propolis silver nano particles and analysis of its antimicrobial properties. J. King Saud Univ. Sci..

[46] Mafra J.F., de Santana T.S., Cruz A.I.C., Ferreira M.A., Miranda F.M., Araújo F.M., Ribeiro P.R., Evangelista-Barreto N.S. (2022). Influence of red propolis on the physicochemical, microbiological and sensory characteristics of tilapia (*Oreochromis niloticus*) salami. Food Chem..

[47] Mizuno S., Miyata R., Mukaide K., Honda S., Sukito A., Sahlan M., Taniguchi T., Kumazawa S. (2021). New compound from the plant origin of propolis from Lombok, Indonesia and its antibacterial activity. Results Chem..

[48] Guzelmeric E., Sipahi H., Özhan Y., Hamitoğlu M., Helvacıoğlu S., Düz G., Akyıldız E., Yaman B.K., Hazar M., Dilsiz S.A. (2023). Comprehensive estrogenic/anti-estrogenic, anticancer, mutagenic/anti-mutagenic, and genotoxic/anti-genotoxic activity studies on chemically characterized black poplar and Eurasian aspen propolis types. J. Pharm. Biomed. Anal..

[49] Brihoum H., Maiza M., Sahali H., Boulmeltout M., Barratt G., Benguedouar L., Lahouel M. (2018). Dual effect of Algerian propolis on lung cancer: Antitumor and chemopreventive effects involving antioxidant activity. Braz. J. Pharm. Sci..

[50] Frion Y., Díaz-García A., Ruiz-Fuentes J., Rodríguez-Sánchez H., Sforcin J.M. (2015). Brazilian green propolis induced apoptosis in human lung cancer A549 cells through mitochondrial-mediated pathway. J. Pharm. Pharmacol..

[51] Teerasripreecha D., Phuwapraisirisan P., Puthong S., Kimura K., Okuyama M., Mori H., Kimura A., Chanchao C. (2012). *In vitro* antiproliferative/cytotoxic activity on cancer cell lines of a cardanol and a cardol enriched from Thai Apis mellifera propolis. BMC Complement. Altern. Med..

[52] Eklund P.C., Backman M.J., Kronberg L., Smeds A.I., Sjöholm R.E. (2007). Identification of lignans by liquid chromatography-electrospray ionization ion-trap mass spectrometry. J. Mass Spectrom..

[53] Lim J.R., Chua L.S., Soo J. (2023). Study of stingless bee (*Heterotrigona itama*) propolis using LC-MS/MS and TGA-FTIR. Appl. Food Res..

[54] Peuhu E., Paul P., Remes M., Holmbom T., Eklund P., Sjöholm R., Eriksson J.E. (2013). The antitumor lignan Nortrachelogenin sensitizes prostate cancer cells to TRAIL-induced cell death by inhibition of the Akt pathway and growth factor signaling. Biochem. Pharmacol..

[55] Li X.-X., Liu C., Dong S.-L., Ou C.-S., Lu J.-L., Ye J.-H., Liang Y.-R., Zheng X.-Q. (2022). Anticarcinogenic potentials of tea catechins. Front. Nutr..

